# Supplementary figures and images for: Ablation of DNA-methyltransferase 3A in skeletal muscle does not affect energy metabolism or exercise capacity
Source: PLoS Genet. 2021 Jan 29;17(1):e1009325. doi: 10.1371/journal.pgen.1009325 (PMC7875352; doi:10.1371/journal.pgen.1009325)

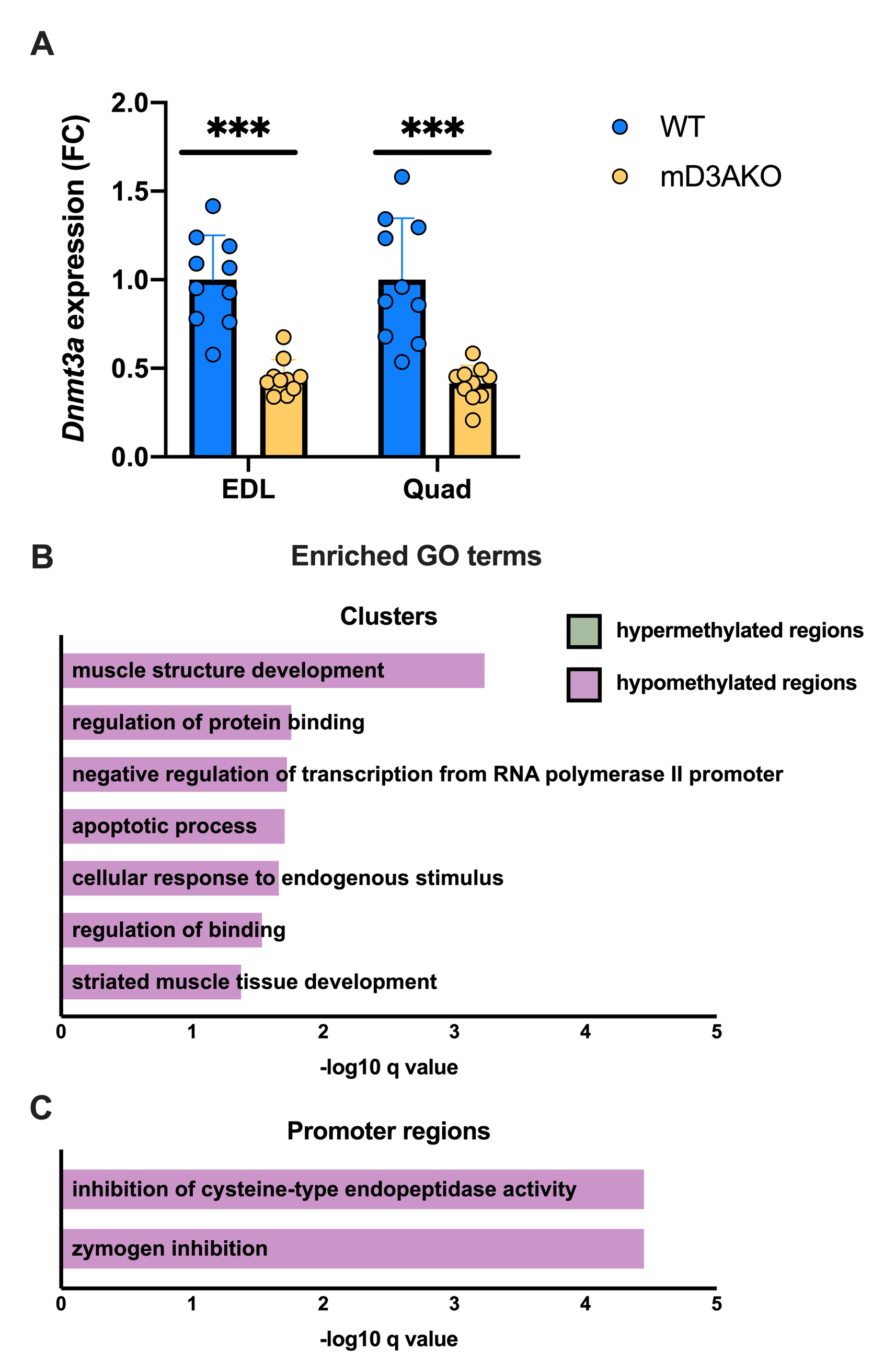

Supplement: S1 Fig — (A) Dnmt3a expression in the EDL and quadriceps muscles of 36-40-week-old male WT and mD3AKO mice compared to the housekeeping gene 18S, fold-change compared to WT, n = 10. Analysed by individual t-tests. Significant GO-BP (gene ontology—biological processes) terms from hypomethylated (B) clusters and (C) promoter regions when comparing mD3AKO to WT soleus muscle. There were no significant terms for hypermethylated regions. FDR 5%. *** P < 0.0005. Data are the mean (bar) with individual data points. (TIF) [file pgen.1009325.s001.tif]

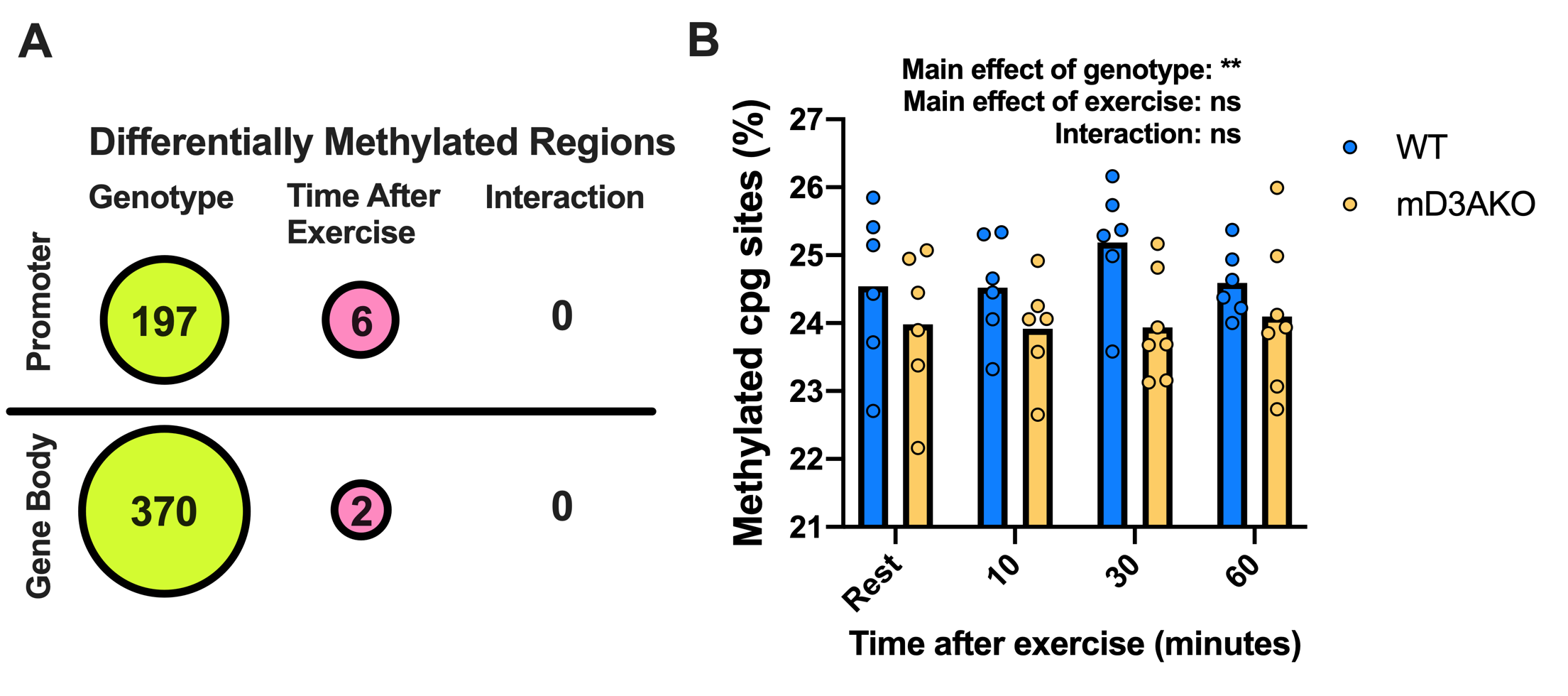

Supplement: S2 Fig — (A) Number of significantly differentially methylated regions in the soleus muscle when comparing genotype, time after exercise and a genotype/exercise interaction. (B) Percentage of total methylated cytosine residues quantified by RRBS in the soleus muscle of non-exercised mice (rest) or taken 10, 30 or 60 minutes after 30 minutes of treadmill running, combined male and female. Analysed by 2-way ANOVA, n = 6–7. Data are the mean (bar) with individual data points. (TIF) [file pgen.1009325.s002.tif]

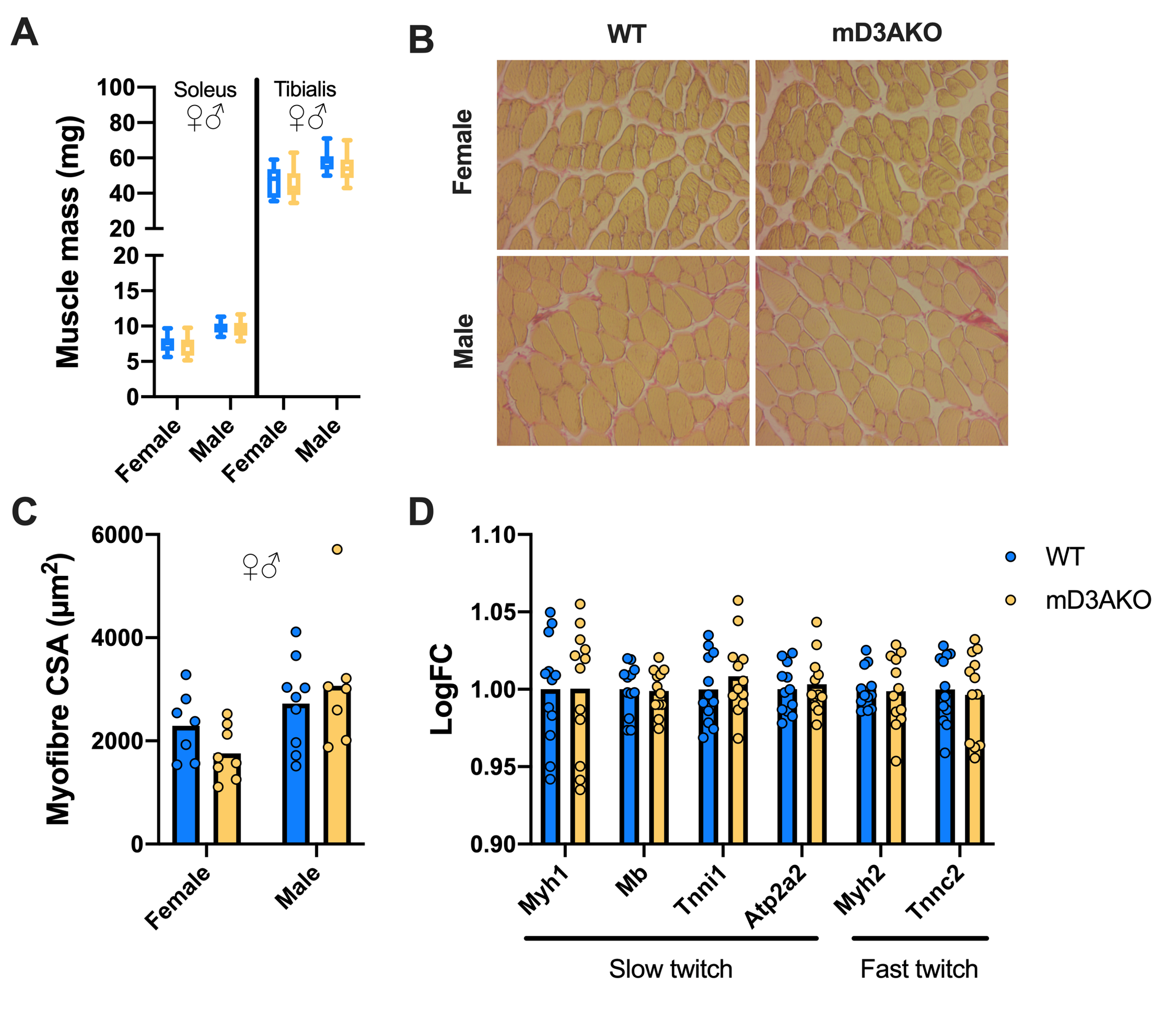

Supplement: S3 Fig — (A) Soleus and tibialis muscle weights of 36–40 week-old male and female WT and mD3AKO mice, n = 15–21. (B) Representative sirius red staining of the plantaris muscle of WT and mD3AKO mice from 12-week-old mice and (C) myofiber cross-sectional area, n = 7–9. Analysed by 2-way ANOVA for a main effect of genotype and a main effect of sex. (D) Gene expression of markers of slow and fast twitch fibers from the soleus muscle of 12-week old mice (male and female combined) normalised to WT, n = 12, analysed by individual t-tests. ♀♂ P < 0.05 main effect of sex. Data are box plots or the mean (bar) with individual data points. (TIF) [file pgen.1009325.s003.tif]

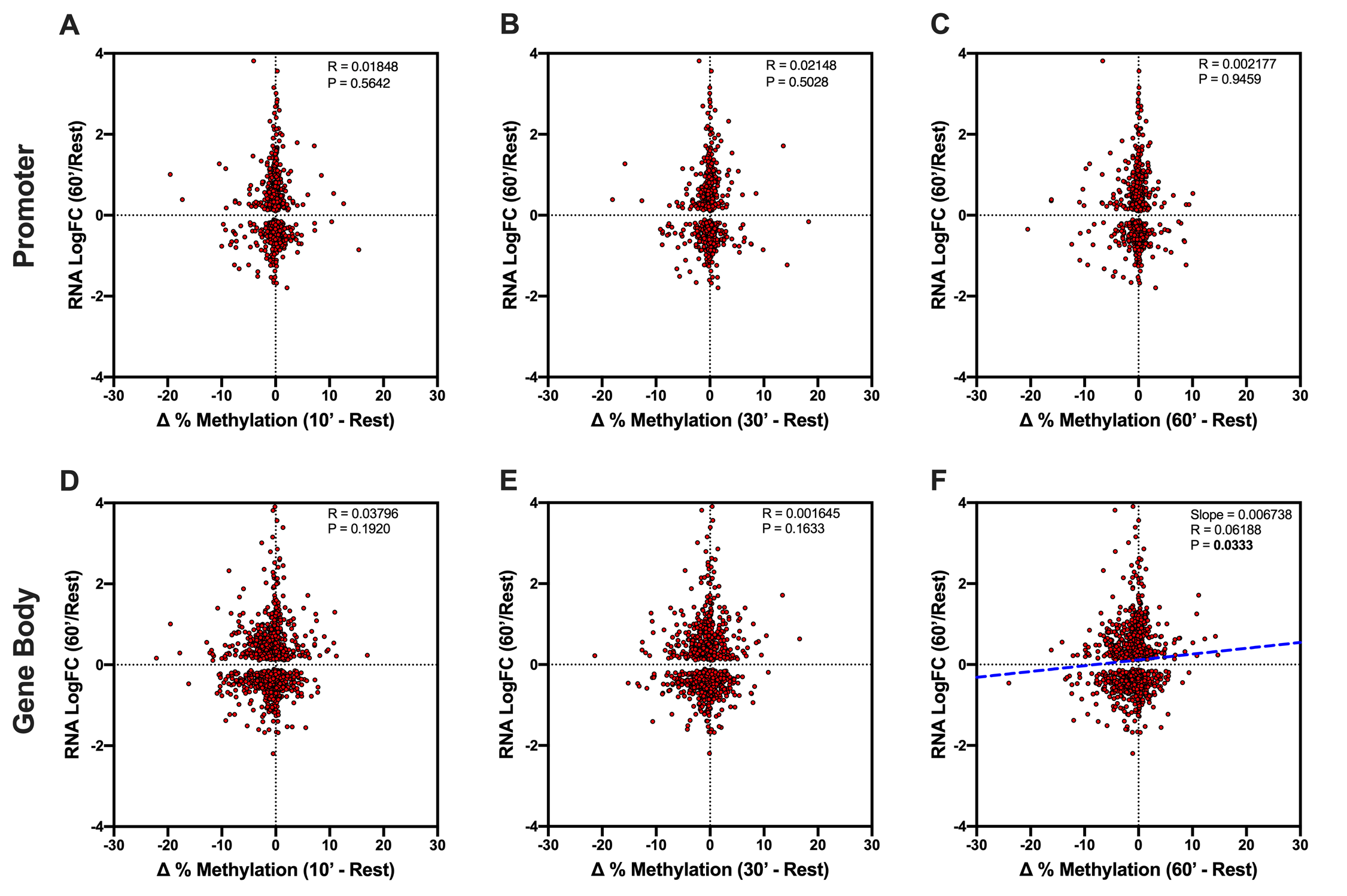

Supplement: S4 Fig — Change in % methylation in promoter regions and gene bodies at (A, D) 10, (B, E) 30 and (C, F) 60 minutes after 30 minutes of treadmill running compared to rested muscle vs LogFC of RNA abundance in muscle 60 minutes after 30 minutes of treadmill running compared to rested muscle. A significant correlation is demonstrated by a blue line. (TIF) [file pgen.1009325.s004.tif]
