# Supplementary material for: Ablation of DNA-methyltransferase 3A in skeletal muscle does not affect energy metabolism or exercise capacity
Source: PLoS Genet. 2021 Jan 29;17(1):e1009325. doi: 10.1371/journal.pgen.1009325 (PMC7875352; doi:10.1371/journal.pgen.1009325)
Supplement: S1 Table — (DOCX) [file pgen.1009325.s007.docx]

**S1 Table. List of primer sequences used in this study**

| **qPCR primers** | | |
| --- | --- | --- |
| **Name** |  | **Sequence (5’ to 3’)** |
| *18S* | F | AGT CCC TGC CCT TTG TAC ACA |
|  | R | GAT CCG AGG GCC TCA CTA AAC |
| *Dnmt3a* | F | CAA GGA AGA GCG CCA GGA G |
|  | R | CTT GGG GGT GTC ACT GCT TT |
| *H19* | F | GAA CAG AAG CAT TCT AGG CTG G |
|  | R | TTC TAA GTG AAT TAC GGT GGG TG |
| *Aldh1l1* | F | CAG GAG GTT TAC TGC CAG CTA |
|  | R | CAC GTT GAG TTC TGC ACC CA |
| *Agtrap* | F | ATG CTT GGG GCA ACT TCA CTA |
|  | R | GCA GCA AGA GAA GGG CTT CA |
| *Pax7* | F | TGG GGT CTT CAT CAA CGG TC |
|  | R | ATC GGC ACA GAA TCT TGG AGA |
| *Eef1a1* | F | ACA CGT AGA TTC CGG CAA GTC |
|  | R | GAT GGT TCG CTT GTC GAT TCC |
| **PCR primers for pyrosequencing** | | |
| **Name** |  | **Sequence (5’ to 3’, bio - biotinylated on 5’ end)** |
| *H19* (gene body) | F | GGT ATT GGG GTA GTA TTG TTA AAG AG |
|  | R | bio - CCT CCT TTC CTA TAA TTC TAT TTC TTT C |
| *Aldh1l1* (promoter) | F | TTA GGT TTA GGG TAT GTT AGG TAA TGA AT |
|  | R | bio - AAA ACT CCC ACT TTC CCT TCT ACA AAA T |
| *Agtrap* (promoter) | F | AGG GTG TTA AAT GGT TTA ATA TTT GG |
|  | R | bio - TTC ATT TCC TAA ATT CTT TTT CTC TCT CA |
| *Pax7* (promoter) | F | GGA GGT AGG TTA TAT TTT TGG AAA TTA TGG |
|  | R | bio - CCT CAA TTC TCT AAA CAC TTT TTC TAT CC |
| *Eef1a1* (promoter) | F | TGG TTG GGA GAG GAA TAT AAT G |
|  | R | bio - CCA AAA AAC CAA CCC AAA AAC A |
| **Sequencing primers for pyrosequencing** | | |
| **Name** |  | **Sequence (5’ to 3’)** |
| *H19* | F | AGT TAT TTT AGG GAT TTT AAA TTAG |
| *Aldh1l1* | F | GGT ATG TTA GGT AAT GAA TTT T |
| *Agtrap* | F | GGT TTA ATA TTT GGG AGT TT |
| *Pax7* | F | ATA TTT TTG GAA ATT ATG GAG A |
| *Eef1a1* | F | ATA TAA TGT TGG GGA TTT TT |
