# Supplementary material for: Ablation of DNA-methyltransferase 3A in skeletal muscle does not affect energy metabolism or exercise capacity
Source: PLoS Genet. 2021 Jan 29;17(1):e1009325. doi: 10.1371/journal.pgen.1009325 (PMC7875352; doi:10.1371/journal.pgen.1009325)
Supplement: Attachment — Submitted filename: Small et al_Reponse to reviewers_R1.docx [file pgen.1009325.s008.docx]

**Response to reviewers**

Comments to the Authors:

**Reviewer #1:** In this study, the authors systematically examine gene expression and DNA methylation with or without exercise in Ck-induced Dnmt3a cKO mice to see the potential function of DNA methylation status in muscle in exercise.

Figure 7 represents the phenotypical difference between WT and KO mice, however, samples of non-exercised ground and exercised group were mixed together for the analysis. The expression and methylation status should be examined separately between WT versus KO at rest and WT versus KO during exercise.

Thank you for this comment. Both transcriptomic and RRBS data were analysed for a main effect of exercise, a main effect of genotype and a genotype/exercise interaction in a 2-way ANOVA type setup. As no interactions were found, figure 7 shows only the effect of genotype and therefore, groups together samples from both exercised and non-exercised mice.

As one of the main aims of this study was to determine differential transcriptomic or DNA-methylation responses to exercise, we believe this is the correct way to analyse this data as it directly tests the interaction between genotype and exercise. We have made this clearer in the statistical analysis section of the methods (lines 549 – 552).

DNA methylation status in skeletal muscle in aging is characterized by overall hypomethylation and partial promoter hypomethylation. Considering that the presented list includes the imprint H19 and Sim2 among the hypomethylated genes, this methylation change may be due to a deletion of the maintenance function of Dnmt3a. This should be disucssed. (The function of Dnmt3a in maintaining DNA methylation, <https://pubmed.ncbi.nlm.nih.gov/19789556/>).

We thank the reviewer for this suggestion and agree with the reviewer that the slight global hypomethylation of the muscle from the mD3AKO animals may be explained by a reduction in the maintenance function of DNMT3a. This may be why the loss of methylation is not more focused on particular regions and is instead found more globally. We have expanded the discussion to consider this point (lines 275-278).

**Reviewer #2:** The authors investigated the function of de novo DNA methylation in fully differentiated skeletal muscle with muscle specific DNA methyltransferase 3A (DNMT3A) knockout mice and investigated the impact of DNMT3A ablation on skeletal muscle DNA methylation, exercise capacity and energy metabolism. The study is very well written and rigorously performed. I very much enjoyed reading the paper and the concept is interesting. The results suggest that use of this model would not unravel the effect of exercise on DNA methylation, hence the focus should perhaps be on human skeletal muscle (single fibre?) models to better understand the effect of exercise on the methylome.

So, overall by reporting these negative results, this is an important report that would add to the current literature.

My minor questions and comments:

1. How is the RNA-seq add to this report if the DNA methylation model did not show direction? Have the transcriptome data was performed at baseline alone? When you stated ‘We
found 8 genes which displayed both significantly altered DNA methylation’ is this after exercise?

We thank the reviewer for his comments. Transcriptomic data was from *soleus* muscle at baseline and 1 hour after exercise and was analysed together for a main effect of exercise, a main effect of genotype or a genotype/exercise interaction. RRBS data was performed on samples taken at baseline or 15, 30 or 60 minutes after exercise. As we found no interactions between exercise and genotype, the transcriptomics and RRBS data discussed in Figure 7 are from both exercised and nonexercised muscle.

After rereading our sentence, we realized that our description of the statistical comparisons was confusing. Therefore, to clarify, we have added information to this sentence (line 223), as well as to the statistical analysis section of the methods (lines 549 – 552).

2. I would add some discussion on the transcriptome at the discussion section.
We agree with the reviewer and we have added additional discussion of the relationship between transcriptomic and methylation data in response to exercise (lines 328-335), as well as validation of methylation and transcriptomic data in other muscle types (lines 343-347).

Regards,
Nir Eynon

**Reviewer #3:**

Summary

The study higlights the impact of skeletal muscle-specific Dnmt3a deficiency on soleus methylation and gene expression, exercise capacity, energy metabolism and whole body metabolism.

The Key findings include:

1. Dnmt3a knock-out soleus muscle display reduced DNA methylation.
2. Muscle-specific Dnmt3a deficiency did not affect exercise capacity or the transcriptional response to acute exercise.

3. mD3AKO mice have normal body composition, whole-body energy expenditure, substrate utilization and glucose tolerance.

4. mD3AKO knockout shows no changes in mitochondrial function but alters transcription of gene(s) involved in skeletal muscle development.

The authors quite nicely performed both methylation and gene expression profiling studies and physiological studies to determine the role of skeletal muscle DNMT3A in exercise and whole-body metabolism. Individual data set is high quality and nicely done, but there are important questions in understanding a whole picture by putting the data together as addressed below.

Major points

1. It is surprising to see that there is an only small negative correlation between differentially methylated genes and differentially expressed genes by Dnmt3a deficiency. What is the correlation of the two by exercise? A poor correlation between the two suggests that methylation and transcriptional changes are not indirect. It might have something to do with the fact that RRBS is biased on the CpG islands. Thus, methylation profile of top Dnmt3a target genes should be examined using bisulfite analysis in soleus and other muscle depot(s).

Thank you for raising this important point. We have included correlations of delta % methylation of differentially methylated regions and logFC RNA expression for genes that were differentially expressed by exercise (See new S4 Fig). Overall, there was a poor correlation between promoter or gene body methylation and transcription of genes that were significantly differentially expressed by exercise at any of the times post exercise. We have added discussion considering these data (lines 328-332).

We agree with the reviewer that as RRBS is heavily biased towards CpG rich regions we may be missing other differentially methylated regions. We have added a sentence to the discussion considering the biases of RRBS (lines 332-335).

We have performed additional experiments in which we did targeted bisulfite pyrosequencing on some of the targets that we identified as both differentially methylated and differentially expressed from the *soleus* RRBS data in the EDL and quadriceps muscle from a separate cohort of mice as well as qPCR of the corresponding transcripts (new figure 8). We found a very good agreement between the RRBS methylation data in *soleus* and targeted pyrosequencing data in EDL and *quadriceps*. This was associated with an increase in expression of the corresponding gene in 3 of the 5 targets we analysed. We have added sentences to the results (lines 235-250) and discussion (lines 343-347) sections considering these data.

We thank the reviewer for this suggestion as we believe it has substantially improved the manuscript, and adds important validation of the RRBS data.

2. Authors should perform some of key analogous studies using other muscles other than soleus muscle. For example, do the authors see the same pattern of regulation in DNA methylation and gene expression of the key gene targets in white or mixed muscles?

Pyrosequencing and qPCR were performed in the EDL and quadriceps of targets genes as discussed above and are depicted in Figure 8.

On the advice of the reviewer we have also added other muscle types to other analyses such as *Dnmt3a* expression after HFD-feeding (Figure 5) and protein abundance of OXPHOS complexes (Figure 6).

3. More thorough model validation is needed for better data interpretation: Authors stated that they used mCK-Cre to avoid developmental effect, as it is expressed quite late during skeletal muscle differentiation and Dnmt3a expression likely comes from satellite cells and non-muscle cells.”. This statement should be revisited as previous literature (doi:10.1016/s1097-2765(00)80155-0; doi:10.1073/pnas.97.7.3467) show that mCK is active embryonically, which could account for the fact that some of developmental genes were altered. Can authors provide evdience that mCK is only active in fully differentiated muscle fiber? mCK is also expressed in heart. Was there any abnormality there? Please show the mRNA and protein level of Dnmt3a KD efficiency in soleus muscle and heart in Fig 1. To claim that they avoided developmental issue, an inducible Cre should be employed.

We agree with the reviewer that mCK is expressed embryonically. However, it is unclear whether there is sufficient mCK expression to pass the threshold needed for efficient Cre recombinase-mediated excision. mCK expression in skeletal muscle is low in the embryo and only rises after birth (<https://doi.org/10.1016/0167-4781(90)90039-5>), and mCK driven tomato fluorescence was only visualised in ~50% of muscle fibers 12 days after birth compared to 100% of fibers 1.5 months after birth ([10.7554/eLife.17355](https://dx.doi.org/10.7554%2FeLife.17355)). However, we can’t rule out the chance of a developmental phenotype and therefore we have altered the manuscript to remove the claim that mCK-Cre model avoids developmental phenotypes. Breeding the mice onto an inducible Cre background is outside the scope of this study and as far as we know, there is no inducible mCK-Cre line and therefore any knockout utilising an inducible HSA or PAX7 promoter may also impact on muscle differentiation from satellite cells in adult animals (i.e. regeneration or hyperplasia).

In regards to evidence that mCK is only active in fully differentiated muscle, we have added data from a time course of *Dnmt3a* expression in primary muscle cells at 0, 1, 3 and 5 days after the initiation of differentiation (new Figure 6E). Only at 5 days of differentiation was there a significant reduction in *Dnmt3a* expression in cells derived from mDA3KO mice. This data agrees with another publication in which the expression of mCK-driven tomato fluorescence was only visible in differentiated myotubes, which were most abundant 5 days after differentiation ([10.7554/eLife.17355](https://dx.doi.org/10.7554%2FeLife.17355)).

We have replaced the DNMT3A tissue abundance western blot (Figure 1A) with a blot that includes heart. All skeletal muscle analysed and heart had a reduction of DNMT3A at the protein level in mD3AKO mice (Figure 1B). We have not performed further analysis on the heart as this investigation was focussed on the role of DNMT3A in skeletal muscle and we were unable to detect any differences in whole-body physiology between WT and mD3AKO mice. We have added a sentence considering this point to the discussion (lines 262-264). We have also added mRNA levels of *Dnmt3a* from EDL and quadriceps of WT and mDA3KO mice showing a similar ~60% reduction in mRNA levels of *Dnmt3a* in these muscles similar to what was seen in the *soleus* (S1 Fig A).

4. There is some inconsistency in choosing muscle depots between experiments. In Fig 6, authors use EDL muscle type to study OXPHOS. But this is a glycolytic white muscle. Since they focused on soleus muscle, the same set of experiments need to be repeated with soleus. In figure 5, authors quadriceps muscles were only looked at. What is the Dnmt3a expression in soleus and EDL?

We agree with the reviewer and have added analysis of additional muscles to be more comprehensive. We have added Western blots of OXPHOS complexes in *soleus* to Figure 6 and have added DNMT3a expression in *soleus* and EDL comparing chow and HFD- fed mice in Figure 5 and have altered the corresponding results sections (lines 196-199 and lines 189-192).

Minor points
1. The gene list of gene ontology data from differentially methylated regions were not provided in Fig. 1.

We have added gene ontology of both differentially methylated clusters and promoter regions (new S1 Fig B-C) which are discussed in the results (lines 119-124).

2. Can authors provide gene ontology analysis of differentially methylated regions by breaking down to hypomethylated and hypermethylated regions?

The new gene ontology analysis added above has been separated into hypo and hyper methylated regions however the only significantly enriched terms were for hypomethylated regions, probably due to the few hypermethylated regions (S1 Fig B-C).

3. In Fig. 4, the authors suggest that male mice have a higher EE compared to females. However, in the treadmill running test, the WT male mice ran for much less distance compared to WT female, which seems counterintuitive. Can the authors provide explanation for that?

Energy expenditure provided in figure 4 was measured in mice housed in metabolic cages (without running wheels) in a TSE indirect calorimetry system and therefore represents 24-hour energy expenditure, not energy expenditure during exercise. We did not measure energy expenditure during exercise however as the reviewer points out, it is likely that female mice would expend more energy as they run for a longer period. We have added this information to the methods to clarify (lines 428-429).

Additionally, energy expenditure was reported as kcal consumed per period (not adjusted to weight), as male mice are heavier and have more lean mass they will expend more energy as shown in Figure 4I.

4. To perform the seahorse assays, authors used muscle satellite cells, which are considered muscle stem or progenitor cells. This should be done with differentiated muscle cells either in vitro or ex vivo.

Seahorse experiments were performed in myotubes differentiated from satellite cells, after 5 days of differentiation. After looking at the methods section we realise this was confusing and have restructured this section to increase clarity (lines 513-516).
